# Supplementary material for: MetaRibo-Seq measures translation in microbiomes
Source: Nat Commun. 2020 Jun 29;11:3268. doi: 10.1038/s41467-020-17081-z (PMC7324362; doi:10.1038/s41467-020-17081-z)
Supplement: Supplementary file 10 — Supplementary Data 7 [file 41467_2020_17081_MOESM10_ESM.zip › File2/Confidence_High_Taxonomy/199344_out.krona.html]

Javascript must be enabled to view this page.

members
magnitude
magnitudeUnassigned
count
unassigned
taxon
rank

199344\_out

148

148
superkingdom
2

976
phylum
148

class
200643
148


SRS049896\_contig\_number\_13314SRS1041142\_contig\_number\_5392SRS1055034\_contig\_number\_6456SRS893383\_contig\_number\_7584
order
171549
4
148

3
family
171550

3
239759
1
genus

SRS077335\_contig\_number\_4217


SRS149181\_contig\_number\_11957
1
species
28117

species
1896969

SRS021948\_contig\_number\_31133
1

1
1853231
family

283168
genus
1


SRS078419\_contig\_number\_22697
1
species
626933

family
171552
6

3
genus
577309

species
1263095

SRS145497\_contig\_number\_12818
1

species
454154
2

SRS049995\_contig\_number\_12618SRS055017\_contig\_number\_31430

3
838
genus


SRS015794\_contig\_number\_8752SRS022609\_contig\_number\_27857
2
species
165179

1

SRS013521\_contig\_number\_3054
1263102
species

2
2005525
family

1
genus
195950

1262979
species

SRS893300\_contig\_number\_119
1

375288
genus
1

1

SRS015782\_contig\_number\_26030
46503
species

2005519
family
93

93
397864
1
genus

SRS097920\_contig\_number\_contig-100\_34430.72793

species
2033407

SRS019445\_contig\_number\_25527SRS024331\_contig\_number\_27935SRS050941\_contig\_number\_11970SRS076756\_contig\_number\_17403SRS076976\_contig\_number\_15109SRS1041140\_contig\_number\_19270SRS104975\_contig\_number\_16202
7


SRS011084\_contig\_number\_22946SRS011302\_contig\_number\_29937SRS013951\_contig\_number\_contig-100\_30293.128555SRS014412\_contig\_number\_14818SRS014415\_contig\_number\_contig-100\_3610.3611SRS015694\_contig\_number\_13622SRS015960\_contig\_number\_21582SRS016018\_contig\_number\_3202SRS016056\_contig\_number\_1160SRS016954\_contig\_number\_18307SRS017307\_contig\_number\_27432SRS017521\_contig\_number\_38874SRS017701\_contig\_number\_11762SRS017821\_contig\_number\_20783SRS018133\_contig\_number\_10363SRS018351\_contig\_number\_9197SRS018936\_contig\_number\_9927SRS019161\_contig\_number\_10164SRS019397\_contig\_number\_8440SRS019968\_contig\_number\_14404SRS020233\_contig\_number\_26451SRS020622\_contig\_number\_5179SRS021484\_contig\_number\_15006SRS022524\_contig\_number\_7893SRS024132\_contig\_number\_30329SRS024435\_contig\_number\_26874SRS045826\_contig\_number\_14355SRS046068\_contig\_number\_1231SRS046369\_contig\_number\_12029SRS047433\_contig\_number\_2189SRS048164\_contig\_number\_14271SRS049773\_contig\_number\_32339SRS050752\_contig\_number\_9617SRS052697\_contig\_number\_24805SRS053356\_contig\_number\_contig-100\_9272.177926SRS053398\_contig\_number\_18232SRS053649\_contig\_number\_13900SRS054059\_contig\_number\_contig-100\_87.45457SRS056273\_contig\_number\_20725SRS058770\_contig\_number\_17423SRS062654\_contig\_number\_19506SRS063040\_contig\_number\_37114SRS063370\_contig\_number\_8617SRS065176\_contig\_number\_4250SRS065504\_contig\_number\_38535SRS075078\_contig\_number\_14828SRS075773\_contig\_number\_34216SRS075821\_contig\_number\_15903SRS077294\_contig\_number\_11981SRS077392\_contig\_number\_39200SRS077552\_contig\_number\_21615SRS077641\_contig\_number\_22243SRS078176\_contig\_number\_12161SRS098644\_contig\_number\_27216SRS098644\_contig\_number\_43009SRS103987\_contig\_number\_31528SRS1041090\_contig\_number\_5517SRS1041143\_contig\_number\_4861SRS105082\_contig\_number\_contig-100\_285.111171SRS1055056\_contig\_number\_11832SRS1055076\_contig\_number\_13670SRS143070\_contig\_number\_contig-100\_3606.78378SRS143148\_contig\_number\_contig-100\_5500.114728SRS143523\_contig\_number\_24898SRS143876\_contig\_number\_3427SRS143895\_contig\_number\_36808SRS144135\_contig\_number\_12459SRS144183\_contig\_number\_14498SRS144603\_contig\_number\_11301SRS146812\_contig\_number\_45756SRS147271\_contig\_number\_27414SRS147557\_contig\_number\_2317SRS147614\_contig\_number\_1342SRS148196\_contig\_number\_28366SRS148511\_contig\_number\_1959SRS148721\_contig\_number\_32026SRS150029\_contig\_number\_15134SRS893170\_contig\_number\_6523SRS893252\_contig\_number\_17104SRS893279\_contig\_number\_10893SRS893327\_contig\_number\_8198SRS893341\_contig\_number\_6466SRS893358\_contig\_number\_9635SRS893378\_contig\_number\_11200SRS893382\_contig\_number\_contig-100\_25649.25650
85
487174
species

39
family
815


SRS012849\_contig\_number\_14598SRS017103\_contig\_number\_15593
39
genus
2
816

1

SRS1041130\_contig\_number\_2453
1262750
species


SRS019381\_contig\_number\_2438
1
species
47678

12

SRS015663\_contig\_number\_25395SRS016267\_contig\_number\_contig-100\_327.83693SRS019787\_contig\_number\_10788SRS020328\_contig\_number\_10566SRS042966\_contig\_number\_18113SRS049402\_contig\_number\_contig-100\_2247.102862SRS049995\_contig\_number\_38277SRS051031\_contig\_number\_24653SRS064757\_contig\_number\_2909SRS100907\_contig\_number\_7363SRS1054716\_contig\_number\_11591SRS140492\_contig\_number\_11434
1262738
species

species
818

SRS140513\_contig\_number\_1197
1

246787
species
1

SRS075341\_contig\_number\_21891

species
310297

SRS043841\_contig\_number\_10526
1

species
1638779

SRS013800\_contig\_number\_13310SRS015369\_contig\_number\_contig-100\_3695.47275SRS016989\_contig\_number\_10808SRS018888\_contig\_number\_1876SRS019910\_contig\_number\_9600SRS024625\_contig\_number\_20919SRS043768\_contig\_number\_25173SRS044535\_contig\_number\_12063SRS050520\_contig\_number\_7SRS054352\_contig\_number\_15820SRS056259\_contig\_number\_2489SRS064557\_contig\_number\_22047SRS077127\_contig\_number\_10731SRS078242\_contig\_number\_20589SRS1041138\_contig\_number\_9294SRS143991\_contig\_number\_10229SRS893342\_contig\_number\_contig-100\_3929.51648
17

28111
species
1

SRS042628\_contig\_number\_23352

species
820

SRS148091\_contig\_number\_9312
1

46506
species
1

SRS098571\_contig\_number\_29451
